# Development of a Geospatial Data-Based Methodology for Stormwater Management in Urban Areas Using Freely-Available Software

**DOI:** 10.3390/ijerph15081703

**Published:** 2018-08-09

**Authors:** Cristina Allende-Prieto, Beatriz I. Méndez-Fernández, Luis A. Sañudo-Fontaneda, Susanne M. Charlesworth

**Affiliations:** 1 Department of Mining Exploitation and Prospecting, University of Oviedo Stormwater Engineering Research Team (UOStormwater), Polytechnic School of Mieres, University of Oviedo, Calle Gonzalo Gutiérrez Quirós s/n, 33600 Asturias, Spain; 2 Department of Construction and Manufacturing Engineering, University of Oviedo Stormwater Engineering Research Team (UOStormwater), Polytechnic School of Mieres, University of Oviedo, Calle Gonzalo Gutiérrez Quirós s/n, 33600 Asturias, Spain; uo139249@uniovi.es (B.I.M.-F.); sanudoluis@uniovi.es (L.A.S.-F.); 3 Centre for Agroecology, Water and Resilience (CAWR), Coventry University, Ryton Gardens, Wolston Lane, Coventry CV8 3LG, UK; s.charlesworth@coventry.ac.uk

**Keywords:** GIS, green infrastructure, GISWATER, land-use planning, LID, lidar data, OSGeo, SDI, SuDS, Stormwater BMP

## Abstract

Intense urbanisation, combined with climate change impacts such as increased rainfall intensity, is overloading conventional drainage systems, increasing the number of combined sewer overflow events and making treatment plants outdated. There is a need for better urban planning, incorporating stormwater and flood management design in order to accurately design urban drainage networks. Geographic Information System (GIS) tools are capable of identifying and delineating the runoff flow direction, as well as accurately defining small-sized urban catchments using geospatial data. This study explores the synergies between GIS and stormwater management design tools for better land-use planning, providing a new methodology which has the potential to incorporate hydraulic and hydrological calculations into the design of urban areas. From data collection to final results, only freely available software and open platforms have been used: the U.S. EPA Storm Water Management Model (SWMM), QGis, PostgreSQL, PostGIS, SagaGIS, and GrassGIS. Each of these tools alone cannot provide all the necessary functionalities for large-scale projects, but once linked to GISWATER, a unique, fast, efficient, and accurate work methodology results. A case study of a newly urbanised area in the city of Gijón (northern Spain) has been utilised to apply this new methodology.

## 1. Introduction

More than half of Earth’s population now lives in urban environments. This change in growth has become a major environmental stressor [1]. Large urban areas have been “waterproofed” by human activities [2], increasing the risk of higher runoff volumes [3], with increased flooding and larger pollutant loads entering water bodies [4]. Runoff in urban areas is transported in the storm sewer system, and with predictions of climate change increasing rainfall intensities and extreme events, drainage systems and treatment plants are at high risk of becoming outdated [5]. Combined sewer overflows (CSOs) are a well-known consequence of such increased surface water flows, leading to the spread of pollution over urbanised areas [6]. Furthermore, there are several practical difficulties related to conventional drainage networks and, more importantly, their integration into models. Watersheds contain a large number of natural and artificial channels (i.e., pipes, culverts, etc.) which can often be difficult to identify and do not usually lend themselves to inclusion in the stream hierarchy of a watershed [7].

Since the early 1970s, developments in hardware and software have led to powerful tools for analysis, management, calculation, monitoring, and interoperability of large amounts of data. Currently, free technologies have a level of development and usability that can exceed private ones, providing useful and user-friendly tools available to all practitioners and academics seeking a professional platform with which to work [8]. The U.S. EPA’s Storm Water Management Model (SWMM) is a good example of a freely available tool used worldwide which can calculate and analyse the hydrologic characteristics of an area and the hydraulics associated with engineering designs [9,10].

Geographic Information Systems (GIS) are software with tools mainly designed to capture, store, manipulate, analyse, manage, and present spatial or geographic data. Within GIS, the portrayal of each type of data can be tailored to meet specific criteria, and the various layers combined to form a single map. They allow the user to obtain a spatial representation of the characteristics of sub-catchments using slope, land-use, or type of vegetation cover by layering with geospatial data [11]. These systems represent a global information resource because the data is georeferenced and there is a process of associating a physical map or raster image with global spatial locations [12]. This framework can be connected with metadata, users, and tools interactively in the Spatial Data Infrastructure (SDI) in order to use these spatial data in an efficient and flexible way. The SDI (*IDEE* as per its Spanish acronym) [13] is a Spanish initiative integrating a wide set of data producers, enabling searching, observation, analysis, and downloading of data as a Web Map Service (WMS) or Web Feature Service (WFS).

Hydrological tools are often combined with digital terrain models available in the GIS software packages, allowing the identification of flows in watersheds, including their main characteristics and properties using geospatial data. As a result, drainage networks and sub-catchments can be accurately delineated [11,14] which is necessary when modelling a hydrological system. However, this has rarely been considered in previous studies using GIS combined with stormwater management design tools [15,16].

Although the state of the art [17,18,19] is based on tools that combine GIS with stormwater management models, the need to obtain reliable input data for the resolution of simulation models makes fieldwork a difficult task in complex urban or peri-urban areas that are difficult to access or to spatially reference. The current trend is the use of data obtained through Light Detection and Ranging (LiDAR), which is both terrestrial (more precise but also more expensive) [20] and aerial [21]. Currently many countries offer aerial LiDAR data through open platforms, such as the United States (NOAA, USGS, and United States Intervention Elevation Inventory), France (GEOSUD, which also offers information from other countries in Asia, Africa, or South America), Mexico (INEGI), and Spain (PNOA, IGN), amongst others [22].

The two main aims of this research were:

1. To develop a new methodology to allow practitioners and academics to combine GIS and stormwater management tools in order to assess the drainage network capacity accurately in urban catchments.
2. To measure the impact on a pre-existing hydrology of rural sub-catchments flowing into urban catchments in areas under development.

The methodology was developed based on freely available software only and applied using a real case study in the city of Gijon (northern Spain).

## 2. Materials and Methods

### 2.1. Location and Main Characteristics for the Case Study: Gijón (Spain)

**Figure 1:**
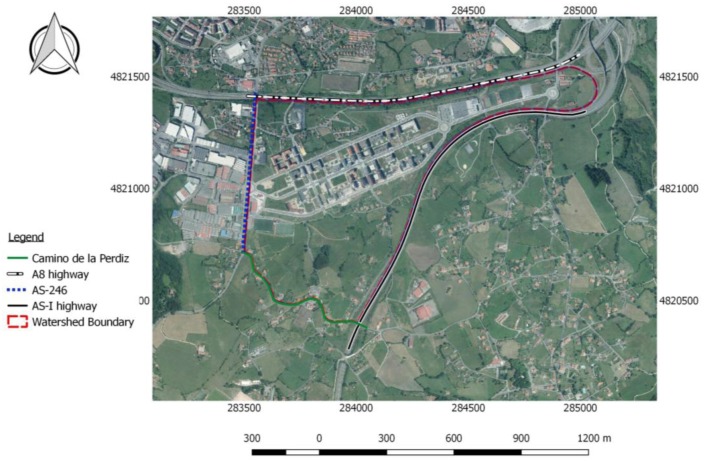
Study catchment at the final stage of development, Gijón, Asturias (Spain).

A newly constructed area located south of the densely urbanised city of Gijón (Asturias, Spain) was chosen due to the observable impact of such development on the hydrology of the area. This area, shown in Figure 1, has gone through several stages of development since 2010 as described below:

- Previous stage. Originally, it was a rural area with agricultural land use until construction began in 2010.
- Development stage. Urban development with an associated increase in population.
- Final stage: Large commercial and service buildings (the neighbourhood has a commercial centre, several football fields and tennis courts, nurseries, and greenhouses, as well as multiple green areas and access roads) (Figure 1).

The study area (1.08 km^2^) has been identified as having potential for growth since the centre of the city is already nearly fully built up, with very little space for further development. In consequence, it is one of the focal points for Gijón’s urban development, urban planning, and mobility plans, where the implementation of new stormwater management methodologies would be required in order to avoid future flooding problems.

The area is surrounded by roads and highways with different traffic intensities (Figure 1):

- East-bound: The AS-I highway
- North-bound: The A8 highway
- West-bound: The AS-246 national road to the west and “El Camino de la Perdiz” to the south.

These roads are higher in altitude than the study area itself, thus they act as barriers preventing surrounding rural areas from contributing to the overall runoff volume of the catchment. Gijón has a Cfb climate (warm temperature, fully humid, with a warm summer) based upon the Köppen–Geiger classification for world climates [23] with an average temperature of 14 °C and 827 mm of annual rainfall. Rainfall patterns in the north of Spain are characterised by mainly being of long duration and low intensity; extreme rainfall intensities do not occur often. In total, 72 years of data were available from the Spanish Meteorological Agency (*AEMET* as per its Spanish acronym), from three meteorological stations in Gijon (Table 1), enabling the maximum daily rainfall to be calculated for several return periods (see Table 2).

Three return periods (2.5, 5, and 10 years), commonly used for design of urban drainage in the north of Spain, were initially selected. Historical rainfall data from these three stations was refined using a Gumbel distribution according to the Spanish standards for superficial drainage [24] and the final results are shown in Table 2.

### 2.2. Experimental Methodology

Soil characteristics for the study area were obtained from the Spanish Ministry of Agriculture, Food and Environment (*MAPAMA* as per its Spanish acronym). In addition, the GIS used was a freely-available software provided by the Open Source Geospatial Foundation (OSGeo) [25]. The OSGeo project developed the following desktop application open source geospatial software: the System for Automated Geoscientific Analyses (SAGA) [26], GRASS [27], PostgreSQL (the most developed object-relational database) [28], PostGIS [29] (a spatial database extender for the PostgreSQL object-relational database), and the QGIS [30] software suite (a geographic information system that integrates PostGIS, SagaGIS, and MapServer).

To relate the water models (the U.S. EPA SWMM [31] and the GIS project (QGIS)), and database storage (PostgreSQL, and PostGIS) the open source software GISWATER [32] was used. This latter software permits the planning of projects in an integrated way by utilizing the advantages of all these technologies.

The identification and delineation of sub-catchments with common characteristics was carried out through stratification with vector and raster geospatial information: urban layout, terrain elevation, land use, and land cover.

Data was obtained from the following sources: *AEMET* [33] for the historical series of meteorological data; the Spanish National Geographic Institute (*IGN* as per its Spanish acronym) for the raster and vector data as well as LiDAR data [34]; *MAPAMA* for the hydrological and subsoil data [35]; the *IDEE* [13], for information related to buildings and ortophotos; and the Gijón Municipal Water Company (EMA, Gijon, Spain) for the GIS data.

**Figure 2:**
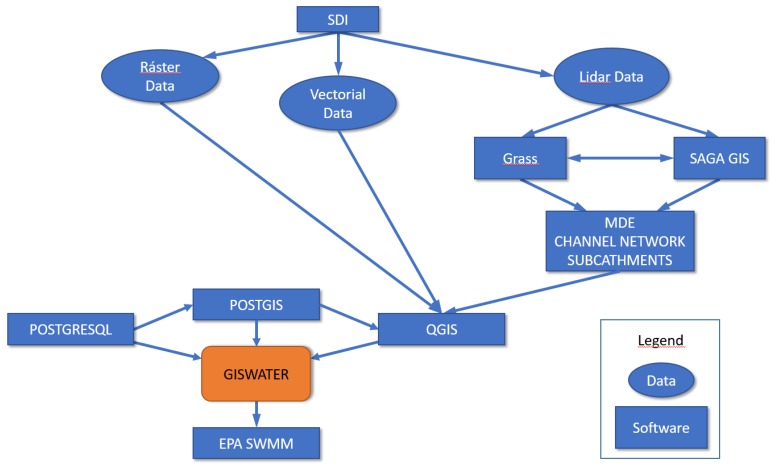
The schematic workflow of this project. SDI: Spatial Data Infrastructure.

A flowchart was generated as shown in Figure 2, which links the software utilised in this research with GISWATER, which was key for this methodology.

**Figure 3:**
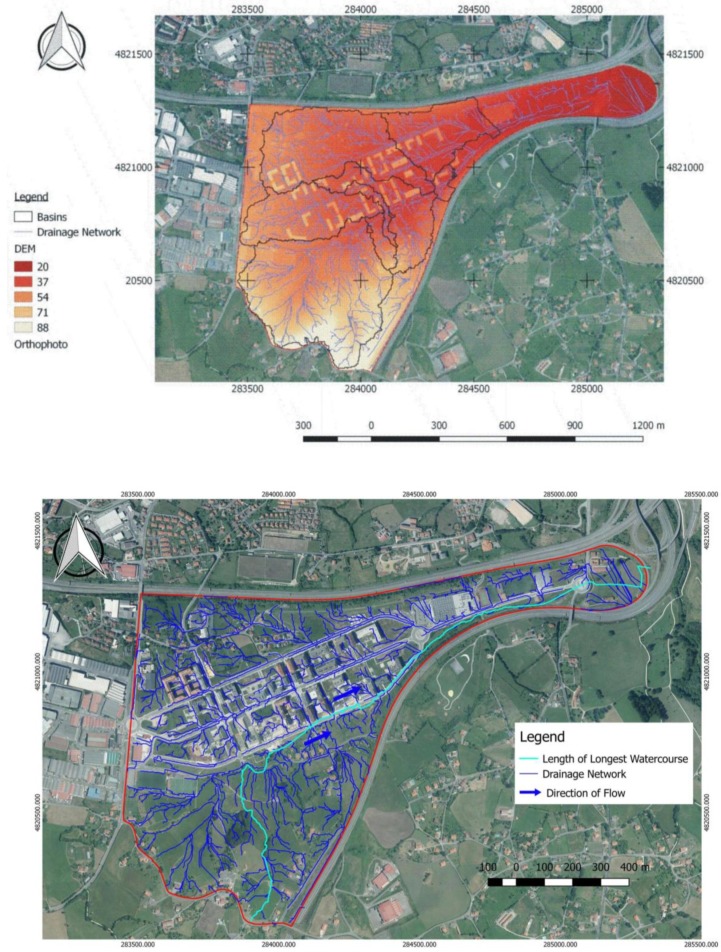
DME with basins and drainage network obtained from hydrological calculations (**Upper image**); and the longest watercourse used to calculate t_c_ in the catchment and drainage network (**Lower image**).

Digital terrain models obtained from point cloud LiDAR data have high-quality terrain information from which drainage networks and watersheds can be extracted. A digital elevation model (DEM) was obtained from a Delaunay triangulation and interpolation from the LiDAR point cloud (Figure 3). Triangular irregular networks (TIN) were connected by processing the triangles to generate drainage networks from sub-catchments.

As seen in Figure 2, the GRASS and SAGA GIS hydrological GIS software were utilised, and 54 catchments were detected. The layer corresponding to soil permeability (geology) was superimposed, obtaining 76 catchments with common slope characteristics and permeability. Finally, these layers were superimposed with land-use information, generating a total of 1398 micro-catchments with the following common characteristics: 752 micro-catchments with permeable soil, 623 micro-catchments with impermeable soil for residential use, and 23 micro-catchments with impermeable soil for industrial use. These data were then inputted into QGis (Figure 2).

**Figure 4:**
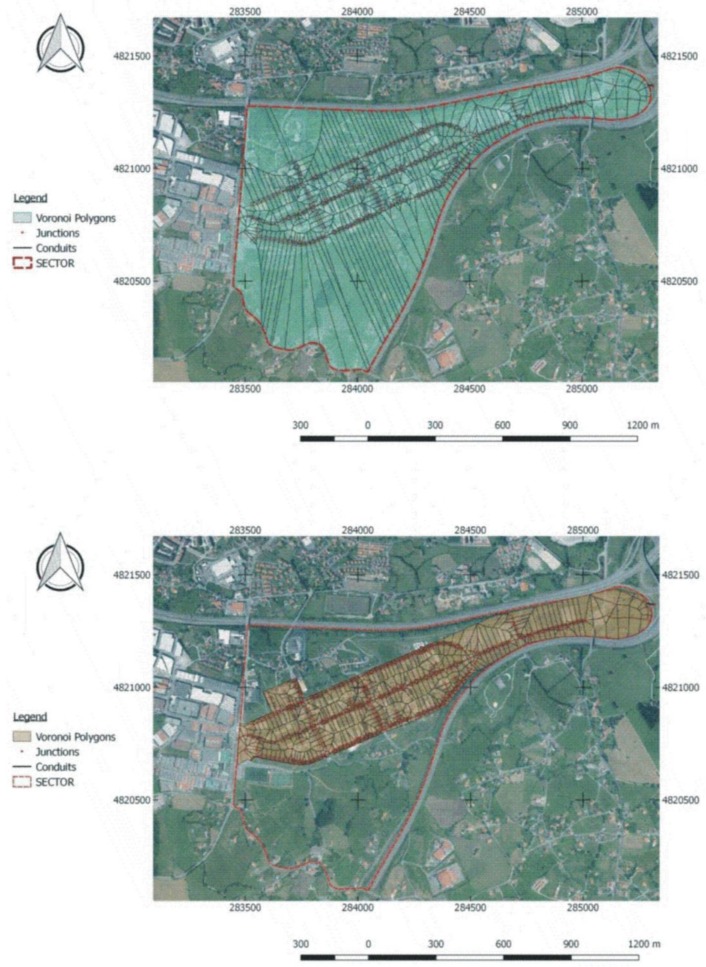
Modelling based on Voronoi polygons for the whole study area (**Upper image**); and Voronoi polygons for the urbanised area in the subcatchment (**Lower image**).

Voronoi polygons were used to calculate runoff flows for each network entry node (Figure 4). The polygons were determined by means of the intersections of bisectors obtained through a method of interpolation based on Euclidean distance, such that the perimeter of each generated polygon was equidistant from its influential node.

Once the sub-catchments had been generated, the data was available for analysis in the stormwater management design tool SWMM.

The rainfall selected for simulation in SWMM was that from “Storm Ana” (18 and 19 December 2017) which was 49.62 mm over a duration of 720 min (12 h) (data obtained from the *AEMET*’s Gijón Meteorological Station, reference number 1208 in Table 1). This rainfall event was similar to the 2.5-year return period storm for Gijón (55 mm in Table 2) and is within the usual return period (2 to 10 years) used to design stormwater systems in urban environments. Also, since it was of long-duration, the concentration time of the catchment could be overcome, reflecting extreme rainfall patterns in the north of Spain. The hyetogram was obtained from the freely-available software “Calculation of the project rain from the procedure of alternating blocks”, designed by the Flumen Institute (Spain) [36] producing a *.dat file that can be imported directly into SWMM. The rainfall event was modelled in SWMM using the Dynamic Wave procedure, as the data recorded were obtained at hourly intervals over long periods. This methodology allowed the evaluation of the hydraulic behaviour of all nodes and drainage conduits from the network.

## 3. Results and Discussion

Once calculations had been carried out, an exportable table containing the results was generated in Excel. Data included: the sub-catchment identifier, the identifier of the node into which the sub-catchment discharged, the rainfall event with which it was associated, sub-catchment area, depth, slope, and length, the identifier associated with snow events (if relevant), percentage of permeable and impermeable surface, and the drainage conduit associated with the node.

**Figure 5:**
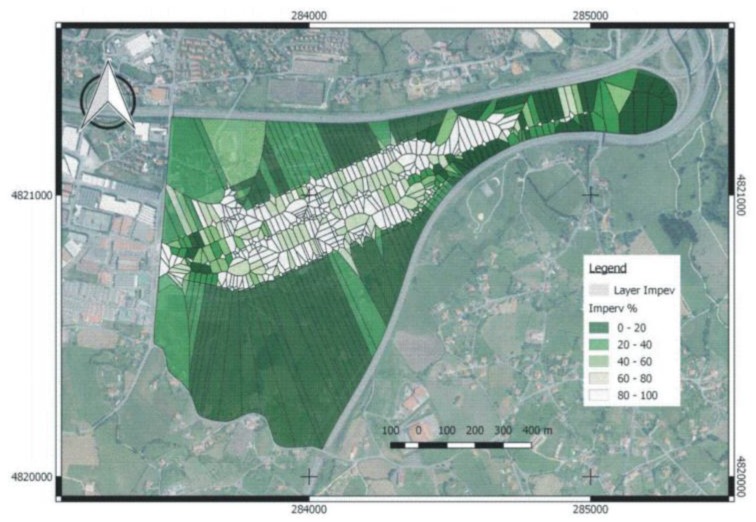
Percentage imperviousness in the catchment.

The percentage of imperviousness in each Voronoi polygon is represented in Figure 5, showing the impact of urbanisation on the catchment.

Hydraulic and hydrological calculations developed in the SWMM provided the following results that were divided into two separate analyses in order to show the impact of rural sub-catchments on the urbanised area:

- A first scenario, taking into account the urbanised area without the rural sub-catchments surrounding it.
- A second scenario, considering the catchment as a whole including adjacent rural areas.

**Figure 6:**
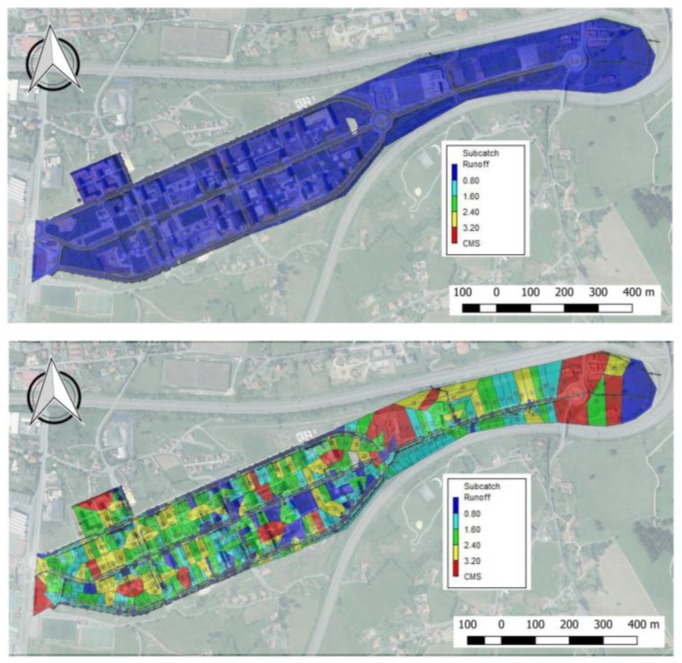
Runoff (m^3^/s or CMS in the legend) from the urbanised catchment during “Storm Ana” from the beginning (**Upper image**) to the end of the storm (**Lower image**).

The first scenario is shown in Figure 6, which illustrates the impact of an extreme rainfall event, particularly at the easterly discharge point of the whole area (Figure 6).

**Figure 7:**
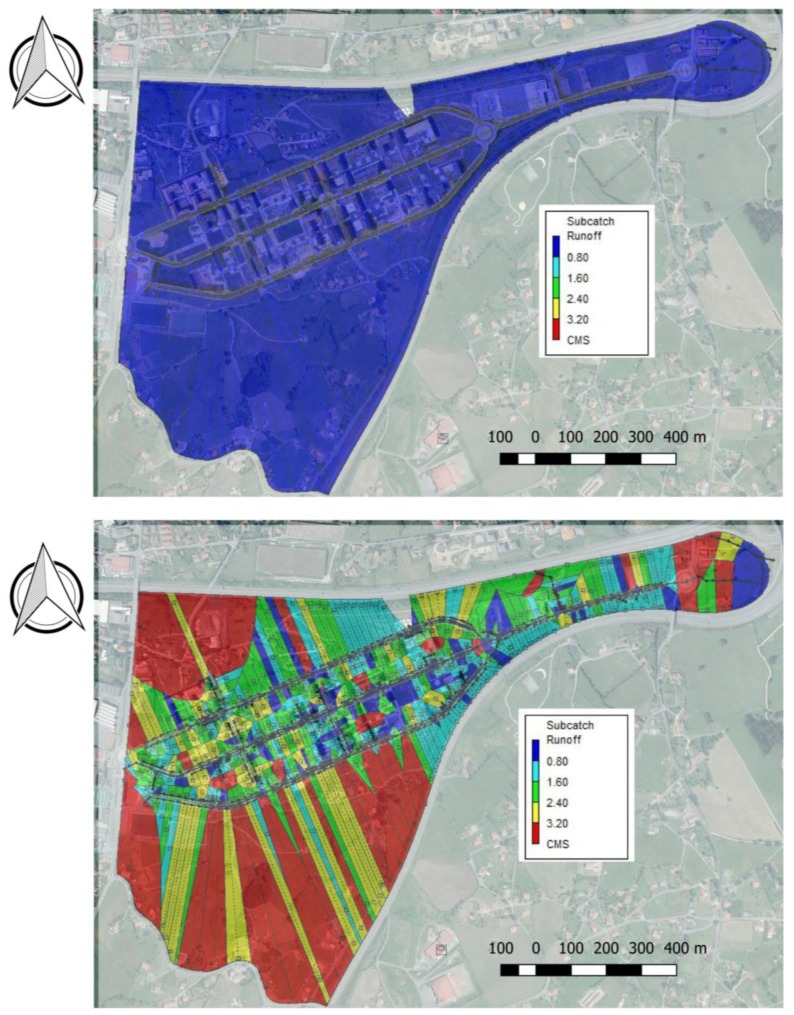
Runoff (m^3^/s or CMS) in the catchment during “Storm Ana” from the beginning (**Upper image**) to the end of the storm (**Lower image**).

From the second scenario, it is possible to assess the significant influence of the rural sub-catchments adjacent to the urbanised area and how they increase the number of areas at risk of flooding (Figure 7). This pattern indicates the need to include adjacent rural sub-catchments in the design considerations for urbanised areas, despite their high permeability, in order to provide more accurate data for the hydraulic design.

## 4. Conclusions

The general findings of this research support those obtained by previous researchers, showing the importance of combining GIS and stormwater management design tools in order to compare the effectiveness of varying stormwater strategies and also to help engineers and planners to better design and assess stormwater management in urban catchments.

However, this research also presents a useful methodology based upon the combination of GIS and freely available stormwater management design tools which can improve accuracy. The results from the case study highlighted the efficiency of this new methodology in assessing hydrological behaviours in urban catchments, and avoided the usual over-engineered hydraulic calculations. This has a large impact on design by avoiding potentially unrealistic claims for flood mitigation by over-engineered drainage systems.

Despite the fact that conventional methods such as topographic surveys or terrestrial LiDAR provide greater precision that those using aerial LiDAR, the latter provided sufficient accuracy to work with scales such as those used in an urban catchment, obtaining high quality results. In addition, it can be downloaded for free in many countries and is relatively simple to use. The information contained in the LiDAR is previously filtered and can be edited according to the needs of the user. The precision obtained in generating the digital model, and the calculation of sub-catchments from input data, has a high level of detail even in urban areas.

The evolution of open source software, which has gained in quality in recent years, provides the practitioner with possibilities of interconnectivity that commercial software cannot. This article has shown that the combination of several types of freely-available software using GISWATER at its core, linking hydraulic analysis programs (such as SWMM) with geographic information systems (such as QGis or SagaGIS) or spatial database managers (such as PostgreSQL or PostGIS) can improve usability, making the results more user-friendly and allowing, in addition, multi-user interactions. Each of these tools alone cannot provide all the necessary functionalities for large-scale projects, but once linked to GISWATER, a unique, fast, efficient, and accurate work methodology results. As a counterpoint, minimum knowledge of each of the platforms is necessary to be able to work with them.

## Figures and Tables

**Table 1:** Meteorological stations used in this study. *AEMET*: Spanish Meteorological Agency (as per its Spanish acronym).

Station Name	*AEMET* Reference	Longitude	Latitude	Altitude (m)	Range of Performance
Gijón La Merced	1208A	5°39′43″ W	43°322L30″ N	22	1 October 1938–31 May 1976
Gijón	1208	5°382L15″ W	43°322L20″ N	3	31 May 1976–5 April 2001
Gijón Musel	1208H	5°412L55″ W	43°332L39″ N	5	5 April 2001–31 December 2011

**Table 2:** Maximum daily rainfall values for each return period.

Maximum Daily Rainfall (mm)	Return Periods
55.00	2.5 years
68.69	5 years
81.09	10 years
